# Reducing the influence of perfectionism and statistics anxiety on college student performance in statistics courses

**DOI:** 10.3389/fpsyg.2022.1011278

**Published:** 2022-10-12

**Authors:** Rongjun Wu, Juan Chen, Qi Li, Hongcang Zhou

**Affiliations:** ^1^School of Applied Meteorology, Nanjing University of Information Science and Technology, Nanjing, China; ^2^Center of Faculty Development and Teaching Evaluation, Nanjing University of Information Science and Technology, Nanjing, China; ^3^Changwang School of Honors, Nanjing University of Information Science and Technology, Nanjing, China

**Keywords:** multidimensional perfectionism scale, statistics anxiety, academic performance, personal standards, teaching innovation

## Abstract

The influence of perfectionism and statistics anxiety on academic performance (AP) in statistics courses was investigated using a multidimensional perfectionism scale and a statistics anxiety rating scale. For perfectionism, the factor of personal standards (PS) had a significant direct positive effect on AP, while the factor of parental expectations (PE) was significantly negatively correlated with AP. Other factors (concern over mistakes, organization, and doubts about actions) did not significantly influence AP. For statistics anxiety, the two factors (test and class anxiety and computation self-concept) significantly impaired AP. These results indicated a need for innovation in classroom instruction and the reform of statistics course content and presentation to reduce statistics anxiety and improve PS. There is also a need to ensure that students better internalize PE and to revise instructional design techniques to enhance students’ independent learning ability.

## Introduction

China noted a rapid socioeconomic development due to its reform and opening-up policy. Meanwhile, China’s higher education is undergoing an unprecedented expansion, which mainly manifested an increase in enrollment of students. As part of the academic training in China’s colleges and universities, students majoring in science and engineering are required to take at least one statistics-related course. These courses are valuable and help to train students in statistical thinking. In addition, these courses will also contribute in quantitative research and comprehension of empirical study. However, many higher education institutions have to cope with high failure rates in statistics-related courses (Smith et al., 2021; Zakariya et al., 2022). This poses a severe challenge to teaching and learning for these courses in higher education.

Some studies were dedicated to improve statistical education, mainly centering on the use of technology, teaching method, and teaching content (Smith, 1998; Bordley and Robert, 2001; Mills, 2002; Zakariya and Bamidele, 2016; Zakariya et al., 2021). The prior researches have played an important role in improving the effectiveness of statistics teaching. However, these teachings were dominated by teacher-directed instruction and note-taking, resulting in learning difficulty for students during class activities (Zakariya et al., 2022). Additional investigations have been focused on non-cognitive characteristics of college students, and showed that attitudes and beliefs toward statistics play an important role in the success or failure of the learning process (Gal and Ginsburg, 1994; Seipel and Apigian, 2005). The findings can help teachers realize what psychological barriers students need to overcome, and then to achieve excellent performance of statistics-related courses.

Perfectionism is an aspect of personality, which can be defined as a complex pattern of deeply embedded psychological characteristics that are largely non-conscious and not easily altered (Habke and Flynn, 2003). So perfectionism is bound to be closely related to academic performance (AP). Meanwhile, statistics-related courses are associated with high levels of anxiety (Chew and Dillon, 2014), which are generally considered to be very difficult in their programs (Onwuegbuzie et al., 2010). Many college students show a high degree of anxiety when faced with the concepts, fundamentals, interpretation, analysis, and application of statistics inherent in such courses (Onwuegbuzie and Wilson, 2003). Particularly, statistics anxiety would result in college students to display feelings of depression, apprehension, anger, worry, panic, and emotionality (Onwuegbuzie, 1997). Therefore, the study of perfectionism and statistical anxiety and their role in statistics-related courses would give teachers an insight into personality traits, allowing to overcome the teaching and learning difficulty and improve AP for statistics.

The intent of the present study is to focus teaching of statistics-related courses on the internal needs of college students. Revealing some inherent behavioral weaknesses of college students during learning statistics-related courses will be helpful for innovation of teaching mode to overcome these weaknesses. Meanwhile, investigation of the role of perfectionism and anxiety in statistics teaching may increase student comprehension, utilization of statistical methods, and AP. The present study is accomplished by (1) analyzing prior literatures and measures of perfectionism and statistical anxiety, and checking the reliability of the measures of perfectionism on college students majoring in science and engineering; and then (2) developing a refined model of perfectionism and statistical anxiety, and their relationship with AP for statistics-related courses, based on a questionnaire survey. Finally, practical implications of analytic results are discussed. The present study will provide guidelines to reform and innovate with statistics teaching in China. It is vital to improve the teaching quality and effectiveness of statistics courses and to implement the concept of student-centered development to increase the quality of talent cultivation.

## Conceptual framework

Perfectionism is a personality trait or cognitive behavioral tendency to pursue perfection in everything. Perfectionism has attracted widespread attention because of its close correlation with disorders such as depression, obsessive-compulsive disorder, and social phobia (Shafran and Mansell, 2001). Most studies of perfectionism have concentrated on distinguishing between the positive and negative aspects of the trait. People who set high standards and allow no mistakes are neurotic perfectionists who experience intense feelings of self-defeat and fear of dysfunction, while a normal perfectionist sets high standards and is able to work hard to complete tasks flexibly in order to pursue self-satisfaction and gain self-esteem (Hamachek, 1978).

The multidimensional perfectionism measures were occurred with the concurrent efforts of many research teams in the 1990s. Two multidimensional perfectionism scale (MPS) instruments independently developed in the 1980s have since been widely used, the Hewitt and Flett MPS (HFMPS) and the Frost MPS (FMPS). Hewitt and Flett (1991) identified three dimensions of perfectionism: self-oriented perfectionism (an individual sets excessively high standards for himself and avoids failure), other-oriented perfectionism (an individual expects others to think well of him), and socially prescribed perfectionism (an individual strives to meet the high expectations of significant others). HFMPS consists of 45 items, with 15 items assigned to each dimension. The six dimensions of FMPS are based mainly on the intrinsic properties of perfectionism and the concerns of perfectionists. The six subscales are concern over mistakes (CM), doubts about actions (DA), personal standards (PS), parental expectations (PE), parental criticism (PC), and organization (OR). CM was regarded as the major dimension in the conceptual framework of perfectionism (Frost et al., 1990).

Most undergraduates tend to exhibit a high level of other-oriented perfectionism, a low level of self-oriented perfectionism, and a high level of socially oriented perfectionism (Walsh and Ugumba-Agwunobi, 2002), and linearly increased levels of perfectionism from 1989 to 2016 was revealed by cross-temporal meta-analysis (Curran and Hill, 2018). Meanwhile, a new generations of college students have more expectations of themselves and others (Curran and Hill, 2018). Previous studies have mainly focused on non-Chinese college students and have investigated the effects of perfectionism on AP in the classroom using HFMPS or FMPS. The instruments used examined the traits of perfectionism and perfectionism in interpersonal relationships. There have been few studies that specifically incorporate characteristics of Chinese culture. Brown et al. (1999) found that the perfectionism factor PS was positively correlated with overall AP, which they attributed to higher PS leading to increased participation in learning and discussion. Bieling et al. (2003) identified that college students with higher levels of perfectionism set themselves higher goals and were more likely to fail, and adaptive perfectionism was moderately associated with AP. These studies have shown that PS were an important mediator between perfectionism and AP. Seipel and Apigian (2005) found that AP was significantly positively correlated with factor PS and significantly negatively correlated with factor OR; other factors of perfectionism, such as CM, did not significantly influence AP but were related to higher levels of anxiety and the perceived level of course difficulty. It appears that the limited research involving college students and statistics-related courses in China has identified a clear link of perfectionism to AP. Therefore, the first research question (RQ) to be investigated in present study is:

RQ1: Are raised PS and increased CM associated with the improvement of AP in China?

There are some definitions with respect to statistics anxiety. Onwuegbuzie (1997) defined statistics anxiety as the fear abandonment that occurs when individuals encounter statistics in whatever form or level. Cruise et al. (1985) defined it as a feeling of anxiety produced when taking a statistics course or gathering, processing, and interpreting data. Furthermore, statistics anxiety is also a form of performance anxiety manifested by disturbing thoughts of worry and mental disorganization, when confronted with statistics content (Zeidner, 1991). In addition, statistics anxiety is also identified as anxiety that arises when confronted with having to learn statistics course content or undertaking statistical analysis and application, and it differs from general anxiety and mathematics anxiety (Siew and McCartney, 2019). Statistics anxiety is a well-documented and common reality for students learning statistics (Onwuegbuzie et al., 2010; Chew and Dillon, 2014), and >80% of college students have experienced various kinds of statistics anxiety and have delayed enrollment in, and therefore taking statistics courses (Onwuegbuzie and Wilson, 2003). Statistics students in online and classroom courses have comparable anxiety levels (Frey-Clark et al., 2019). Statistics courses are regarded as the most anxiety-inducing course in college student curriculums (Chew and Dillon, 2014). Some studies showed that statistics anxiety accompanied with negative attitudes and poor mathematical foundations resulted in low AP in introductory statistics courses (Chiesi and Primi, 2010). Statistics anxiety was associated with test and class anxiety (TCA) (Macher et al., 2013), and anxiety experienced by graduate students when writing research reports (Onwuegbuzie, 1997). Thereby, statistics anxiety significantly affected AP (Macher et al., 2013) and high school math matriculation scores (Zeidner, 1991), and hindered performance for approximately 80% of graduate students in research methodology course (Onwuegbuzie and Wilson, 2003).

Despite lots of researches on the influence of statistics anxiety on AP, there is no other reliable psychometrically valid assessment tool that exclusively assesses statistics anxiety of students, except the statistical anxiety rating scale (STARS) and statistical anxiety scale (SAS) (O’Bryant et al., 2021). The scores produced from the instrument of STARS would help instructors better understand statistics anxiety for college students, and implement valuable instructional methods to enhance AP in statistics-related courses in China. Therefore, the second RQ to be investigated in present study is:

RQ2: How great are the effects of statistics anxiety and self-perception of statistics on AP in China?

Perfectionism may lead to improved performance, and PS and OR are positive characteristics of perfectionism. However, it can also result in higher anxiety, setting of unattainable goals, and procrastination (Frost et al., 1993; Enns and Cox, 1999). So there has been a clear link between statistics anxiety and perfectionism among college students. Perfectionists with higher levels of other-oriented or socially oriented perfectionism tend to have higher levels of statistics anxiety accompanied by complex emotional responses and a greater tendency to resist learning (Onwuegbuzie and Daley, 1999). Walsh and Ugumba-Agwunobi (2002) found that Self-oriented perfectionism among undergraduate students was associated with statistics anxiety, even though controlling the anxiety and procrastination.

Perfectionism has been shown to exist among college students (Seipel and Apigian, 2005). Given that perfectionism is a relatively unchangeable personality of students, which is associated with factors that influence AP, it would be beneficial to reveal the direct implications to AP, and to improve effectiveness of statistics instruction. Moreover, little study has been conducted to cope with the psychological barriers and anxiety preventing students from achieving good AP in statistics-related courses.

In order to effectively innovate with and reform statistics teaching to make it more student-centered, it is necessary to clearly understand the psychological characteristics of college students who enroll in statistics-related courses in China. The present study intends to investigate perfectionism and statistics anxiety in college students taking statistics-related courses and the effects of perfectionism and statistics anxiety on student AP.

## Materials and methods

### Participants

The participants in the survey were majors in ecology, agricultural resources and environment, applied meteorology, and environmental science and engineering programs at Nanjing University of Information Science and Technology. We received 212 valid FMPS questionnaires from sophomores surveyed who were enrolled in Biostatistics or Probability and Mathematical Statistics courses (84 male and 128 female, mean age = 19.04, SD = 1.35), and 301 valid STARS questionnaires were collected from the statistics anxiety survey (134 male and 167 female, mean age = 19.54, SD = 1.89).

### Description of the Frost multidimensional perfectionism scale and statistical anxiety rating scale instruments

#### Chinese version of Frost multidimensional perfectionism scale

The Chinese version of FMPS we used was a version revised by Zi and Zhou (2006) from the University of Hong Kong version devised by Cheng et al. (1999). It is similar to the original English version of FMPS, has satisfactory reliability and validity, and is suitable for use in a Chinese cultural environment (Zi and Zhou, 2006). The five dimensions of the Chinese version of FMPS are CM, DA, PS, PE and, OR. The Chinese version of FMPS totaled 27 items (Table 1) and omitted the dimension of PC found in the original FMPS. Each item was rated on a scale of 1–5 (strongly disagree–strongly agree). The subjects rated each statement based on their own reality, with higher scores indicating greater perfectionism.

**TABLE 1:** Items and their subscale attribution on FMPS (Frost et al., 1990) and Chinese version FMPS (Zi and Zhou, 2006).

Item	Question	FMPS	Chinese version FMPS
Q1	My parents set very high standards for me	PE	PE
Q2	Organization is very important to me	OR	OR
Q3	As a child, I was punished for doing things less than perfect	PC	
Q4	If I do not set the highest standards for myself, I am likely to end up a second rate person	PS	PS
Q5	My parents never tried to understand my mistakes	PC	
Q6	It is important to me that I am thoroughly competent in everything I do	PS	
Q7	I am a neat person	OR	OR
Q8	I try to be an organized person	OR	OR
Q9	If I fail at work/school, I am a failure as a person	CM	CM
Q10	I should be upset if I make a mistake	CM	
Q11	My parents wanted me to do the best at everything	PE	PE
Q12	I set higher goals than most people	PS	PS
Q13	If someone does a task at work/school better than I, then I feel like I failed the whole task	CM	CM
Q14	If I fail partly, it is as bad as being a complete failure	CM	CM
Q15	Only outstanding performance is good enough in my family	PE	
Q16	I am very good at focusing my efforts on attaining a goal	PS	
Q17	Even when I do something very carefully, I often feel that it is not quite right	DA	DA
Q18	I hate being less than the best at things	CM	PS
Q19	I have extremely high goals	PS	PS
Q20	My parents have expected excellence from me	PE	PE
Q21	People will probably think less of me if I make a mistake	CM	CM
Q22	I never felt like I could meet my parent’s expectations	PC	
Q23	If I do not do as well as other people, it means I am an inferior human being	CM	CM
Q24	Other people seem to accept lower standards from themselves than I do	PS	PS
Q25	If I do not do well all the time, people will not respect me	CM	CM
Q26	My parents have always had higher expectations for my future than I have	PE	PE
Q27	I try to be a neat (tidy) person	OR	OR
Q28	I usually have doubts about the simple everyday things I do	DA	DA
Q29	Neatness is very important to me	OR	OR
Q30	I expect higher performance in my daily tasks than most people	PS	PS
Q31	I am an organized person	OR	OR
Q32	I tend to get behind in my work because I repeat things over and over	DA	DA
Q33	It takes me a long time to do something “right”	DA	DA
Q34	The fewer mistakes I make, the more people will like me	CM	
Q35	I never felt like I could meet my parent’s standards	PC	PC

#### Statistical anxiety rating scale

Statistical anxiety rating scale (Cruise et al., 1985) is a widely used instrument to measure statistics anxiety in students (Chew and Dillon, 2014). STARS consists of six dimensions of statistics anxiety that were identified by confirmatory factor analysis (CFA), worth of statistics (WS), interpretation anxiety (IA), TCA, computation self-concept (CS), fear of asking for help (FAH), and fear of statistics teachers (FST). Vigil-Colet et al. (2008) observed that among the six factors of STARS, only three factors (TCA, FAH, and IA) were directly related to statistics anxiety, while the other three were related to student self-perception. The TCA factor indicates anxiety generated when attending a statistics class or taking a statistical test; the FAH factor indicates anxiety generated when asking classmates or teachers for help in understanding the content of the class or other statistical concepts; the IA factor indicates anxiety generated when making decisions based on or interpreting statistical data (Cruise et al., 1985). Most of the items indicating IA are related to the application of statistics in scientific research.

We analyzed only the factors of TCA and FAH as indicators of statistics anxiety since the subjects were undergraduate students. We analyzed the three self-perception factors (WS, FST, and CS) as they indicated student perceptions of the usefulness of statistics, the sympathy of statistics instructors, and their own ability to solve mathematical problems (Cruise et al., 1985). Table 2 shows the list of items from the original STARS (Cruise et al., 1985) and the five grouped factors mentioned above. The 51 items of the STARS instrument were scored on a five-point Likert scale and were grouped into two parts, consistent with Cruise et al. (1985) and Siew and McCartney (2019). The first part asks respondents to rate their level of anxiety in the situation described on a scale from 1 to 5 (no anxiety–a great deal of anxiety), and the second part asks them to rate their level of agreement with statements related to statistics on a scale from 1 to 5 (strong disagreement with statement–strong agreement with statement).

**TABLE 2:** Items from the statistical anxiety rating scale and grouped by Factor of test and class anxiety (TCA), fear of asking for help (FAH), worth of statistics (WS), computation self-concept (CS), and fear of statistics teachers (FST), originated from Cruise et al. (1985) and Siew and McCartney (2019).

Item	Question	Factor
Q1	Studying for an examination in a statistics course	TCA
Q4	Doing the homework for a statistics course	TCA
Q8	Doing the final examination in a statistics course	TCA
Q10	Walking into the classroom to take a statistics test	TCA
Q13	Finding that another student in class got a different answer than you did to a statistical problem	TCA
Q15	Waking up in the morning on the day of a statistics test	TCA
Q21	Enrolling in a statistics course	TCA
Q22	Going over a final examination in statistics after it has been graded	TCA
Q3	Going to ask my statistics teacher for individual help with material I am having difficulty understanding	FAH
Q16	Asking one of your lecturers for help in understanding a printout	FAH
Q19	Asking someone in the computer center for help in understanding a printout	FAH
Q23	Asking a fellow student for help in understanding a printout	FAH
Q24	Since I am by nature a subjective person, the objectivity of statistics is inappropriate for me	WS
Q26	I wonder why I have to do all these things in statistics when in actual life I’ll never use them	WS
Q27	Statistics is worthless to me since it’s empirical and my area of specialization is philosophical	WS
Q28	Statistics takes more time than it’s worth	WS
Q29	I feel statistics is a waste	WS
Q33	I lived this long without knowing statistics, why should I learn it now?	WS
Q35	I don’t want to learn to like statistics	WS
Q36	Statistics is for people who have a natural leaning toward math	WS
Q37	Statistics is a grind, a pain I could do without	WS
Q40	I wish the statistics requirement would be removed from my academic program	WS
Q41	I don’t understand why someone in my field needs statistics	WS
Q42	I don’t see why I have to clutter up my head with statistics. It has no significance to my life work	WS
Q45	I can’t tell you why, but I just don’t like statistics	WS
Q47	Statistical figures are not fit for human consumption	WS
Q49	Affective skills are so important in my profession that I don’t want to clutter my thinking with something as cognitive as statistics	WS
Q50	I am never going to use statistics so why should I have to take it?	WS
Q25	I haven’t had math for a long time. I know I’ll have problems getting through statistics	CS
Q31	I can’t even understand seventh- and eighth-grade math; how can I possibly do statistics?	CS
Q34	Since I have never enjoyed math I don’t see how I can enjoy statistics	CS
Q38	I don’t have enough brains to get through statistics	CS
Q39	I could enjoy statistics if it weren’t so mathematical	CS
Q48	Statistics isn’t really bad. It’s just too mathematical	CS
Q51	I’m too slow in my thinking to get through statistics	CS
Q30	Statistics teachers are so abstract they seem inhuman	FST
Q32	Most statistics teachers are not human	FST
Q43	Statistics teachers talk a different language	FST
Q44	Statisticians are more number oriented than they are people oriented	FST
Q46	Statistics teachers talk so fast you cannot logically follow them	FST

Respondents were also asked to provide student ID and specify their gender and birthplace (urban or rural) in the questionnaire in keeping with the research objectives. The student ID allowed us to obtain the final grades the student obtained in statistics courses (Biostatistics, Probability, and Mathematical Statistics) for analysis. The *t*-test of group data showed that there was no significant difference between the scores of Biostatistics and those of Probability and Mathematical Statistics (*t* = 0.841, *p* = 0.20 > 0.05).

### Statistical analysis

Reliability and validity tests of the scales, correlation analysis, Mann–Whitney *U*-tests, independent sample *T*-tests, and CFA were performed using SPSS statistical software (version 16.0). Structural equation models (SEM) with maximum likelihood estimation, implemented in AMOS 21, IBM SPSS, were used to analyze the relationship between perfectionism and course performance.

Several indices of fit were used to evaluate the models. A goodness-of-fit index (GFI) quantifies the proportion of variance accounted for by comparing the sample covariance matrix with the estimated population covariance matrix (Shevlin and Miles, 1998). A comparative fit index (CFI) was used to compare the hypothesized model against an independence model (Bentler, 1988). The Tucker–Lewis index (TLI) is a CFI that was used to compare models in different samples of unequal size (Tucker and Lewis, 1973). The incremental fit index (IFI) measures the degree to which a model improves data fitting (Fan and Wang, 1998; Cox et al., 2002). The root mean square error of approximation (RMSEA) was used to estimate the amount of model misfit (Fan and Wang, 1998; Cox et al., 2002). The following frequently used criteria were taken to evaluate model adequacy: GFI > 0.80, CFI > 0.90, TLI > 0.90, IFI > 0.90, and RMSEA < 0.08 (Arpaci and Baloğlu, 2016). No item was allowed to load on more than one factor. Correlated errors were not specified in the models.

## Results

### Reliability and validity test of the scales

#### Chinese version of Frost multidimensional perfectionism scale

The Kaiser–Meyer–Olkin (KMO) test and Bartlett’s sphericity test were conducted on the FMPS questionnaire data using SPSS 16.0. The KMO statistic was 0.848 (>0.7), which indicated that the correlation matrix had common factors and factor analysis can be carried out. The Bartlett’s sphericity test result (χ^2^ = 2,613, *df* = 351, *p* < 0.001) indicated that each factor was not independent and that common factors could be extracted suitable for exploratory factor analysis. Factor analysis was conducted to obtain five factors with eigenvalues >1 that explained 60.34% of the total variance (>50%). The results showed that the factors of the items could be extracted. Combined with the analysis of the scree plot, we found that the questionnaire structure and item distribution were most effective when the five factors were extracted. Varimax orthogonal rotation was used for factor rotation. The standard loading, eigenvalue, and variance contribution of each factor are shown in Table 3. After rotation, we found that CM, OR, PS, PE, and DA, respectively explained 14.95, 14.71, 11.41, 10.25, and 9.03% of the total variance.

**TABLE 3:** Total variance explained by revised Chinese FMPS (FMPS-I) obtained by principal component analysis and varimax orthogonal rotation.

Factors and items in FMPS-I	Factors					Original FMPS	Chinese FMPS
			
	1	2	3	4	5		
Concern over mistakes (CM)							
Q23	**0.803**	−0.032	0.045	0.046	0.216	CM	CM
Q25	**0.708**	−0.001	0.219	0.061	0.190	CM	CM
Q13	**0.701**	−0.055	0.304	0.080	−0.002	CM	CM
Q21	**0.673**	−0.049	0.024	0.364	0.145	CM	CM
Q9	**0.667**	−0.050	0.263	0.118	0.117	CM	CM
Q14	**0.621**	−0.090	0.256	0.000	0.289	CM	CM
Q35	0.606	−0.206	−0.140	0.401	0.092	PC	PC
Organization (OR)							
Q29	−0.091	**0.855**	−0.055	0.042	0.155	OR	OR
Q7	−0.087	**0.795**	−0.162	−0.033	0.022	OR	OR
Q27	0.005	**0.788**	0.002	−0.042	0.215	OR	OR
Q31	−0.062	**0.736**	0.193	0.113	−0.142	OR	OR
Q8	−0.038	**0.714**	0.163	0.038	0.019	OR	OR
Q2	−0.001	**0.641**	0.257	0.031	−0.205	OR	OR
Q30	−0.029	0.537	0.417	0.001	0.241	PS	PS
Personal standards (PS)							
Q19	0.178	0.253	**0.790**	0.086	−0.019	PS	PS
Q12	0.169	0.153	**0.776**	0.125	−0.045	PS	PS
Q24	0.264	0.020	**0.620**	−0.038	0.218	PS	PS
Q4	0.143	−0.026	**0.612**	0.293	0.181	PS	PS
Q18	0.435	0.139	0.445	−0.002	0.356	CM	PS
Parental expectations (PE)							
Q1	0.031	−0.026	0.123	**0.780**	−0.146	PE	PE
Q20	0.016	0.171	0.171	**0.733**	0.179	PE	PE
Q11	0.189	0.045	0.117	**0.719**	0.108	PE	PE
Q26	0.388	−0.011	−0.081	**0.710**	0.146	PE	PE
Doubts about actions (DA)							
Q28	0.232	−0.090	0.210	0.135	**0.683**	DA	DA
Q32	0.158	0.050	−0.083	0.323	**0.680**	DA	DA
Q33	0.230	0.161	0.044	0.019	**0.677**	DA	DA
Q17	0.261	0.054	0.386	−0.167	**0.593**	DA	DA
Eigenvalue	6.648	4.297	2.281	1.822	1.244	Extraction sums of squared loadings	
% of variance	24.622	15.915	8.447	6.750	4.607		
Cumulative%	24.622	40.537	48.984	55.734	60.341		
Eigenvalue	4.037	3.972	3.080	2.767	2.437	Rotation sums of squared loadings	
% of variance	14.951	14.711	11.406	10.248	9.025		
Cumulative%	14.951	29.662	41.068	51.316	60.341		

The bold values denote the items for which the loading of two or more latent factors >0.40.

Using Table 3, we deleted items for which the loading of two or more latent factors >0.40 (Q35, Q18, and Q30). The final MPS that we used consisted of five factors and 24 items. We refer to this version of FMPS as the revised Chinese version of FMPS, FMPS-I, which had three items less than the original Chinese version of FMPS and 11 items and one factor less than the original FMPS. The five factors that we obtained by exploratory factor analysis were very similar to the five dimensions of the Chinese version of FMPS and to five of the six dimensions of the original FMPS. Principal component analysis and varimax orthogonal rotation of the final 24 items showed that 15.22, 14.79, 11.22, 11.08, and 9.61% of the total variance was, respectively explained by CM, OR, PS, PE, and DA. The five factors together explain 61.93% of the total variance, and the results we obtained support the accuracy of the theoretical conception of the instrument and the structure of the scale used.

The reliability of the questionnaire was tested using the responses of the sample of 212 college students. The overall Cronbach’s α coefficient for the entire questionnaire was 0.854, indicating good internal consistency and high reliability. Cronbach’s α coefficients for CM, OR, PS, PE, and DA were 0.852, 0.857, 0.777, 0.783, and 0.714 (>0.7), which indicates that each factor had acceptable and reliable internal consistency.

#### Statistical anxiety rating scale

We analyzed only the statistics anxiety and self-perception scales. The KMO test statistic for statistics anxiety was 0.942 (>0.7), which indicates that there were common factors in the correlation matrix, and the questionnaire was effective. Bartlett’s sphericity test gave χ^2^ = 3,118 (*df* = 66, *p* < 0.001), and the cumulative variance with varimax orthogonal rotation was 74.321%. The KMO test statistic for the self-perception scale was 0.982 (>0.7), which indicates that there were common factors in the correlation matrix. Bartlett’s sphericity test gave χ^2^ = 10,640 (*df* = 378, *p* < 0.001), and the cumulative variance with varimax orthogonal rotation was 77.80%.

The reliability test of the questionnaire used the responses of 301 college students. The results showed that overall Cronbach’s α for the questionnaire was 0.961, which indicates good internal consistency and high reliability. Cronbach’s α coefficients for statistics anxiety and self-perception were 0.957 and 0.982, both above the criterion of 0.70, indicating that the scale had acceptable internal consistency and reliability for each dimension.

### The relationship between perfectionism and academic performance

We performed the Mann–Whitney *U*-test on the scores for each factor of perfectionism grouped by gender. The scores of female students for factor PE were significantly less than those of male students (*Z* = −3.326, *p* = 0.002), but there was no significant difference between genders for other factors. There were also no significant differences between genders in the total perfectionism scores for all factors (*Z* = −1.459, *p* = 0.165); nor were there significant differences for all factors between rural and urban birthplaces when students were grouped by place of birth (*p* > 0.025). Gender and place of birth therefore have little explanatory power for individual differences in perfectionism. The only factor that has explanatory power is PE. There are lower PE for female students than for male students.

Confirmatory factor analysis was conducted on the five factors CM, OR, PS, PE, and DA as well as the 24 items determined by maximum likelihood estimation. The results showed that the fitting index was relatively satisfactory with χ^2^/*df* = 1.793 (<2). The GFI, Bentler’s CFI and the IFI were 0.895, 0.923, and 0.931, which were all around 0.90. The RMSEA was 0.051 (<0.08), which indicates that the model could be acceptable. The factor loading of each item was between 0.52 and 0.89, which indicates good reliability. The preceding indexes indicate that our structural model is stable and reliable.

**FIGURE 1:**
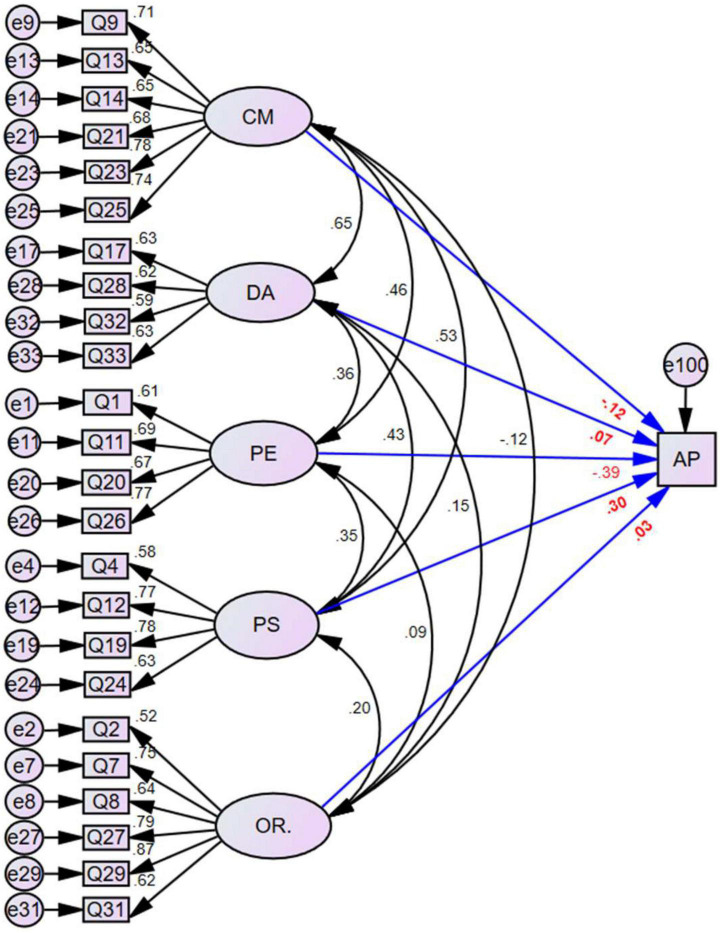
Complete SEM-FMPS-I (revised Chinese) model structure showing the relationships between factors of perfectionism and academic performance. CM, concern over mistakes; DA, doubts about actions; PE, parental expectations; PS, personal standards; OR, organization; AP, academic performance in statistics courses.

We created a preliminary structural equation model (SEM-FMPS-I; Figure 1) using the acceptable model consisting of five latent factors and 24 items. We investigated the effects of the latent factors of perfectionism on AP in statistics courses, with AP as the dependent variable and CM, OR, PS, PE, and DA as independent variables. SEM-FMPS-I allowed for pairwise correlations among the independent variables. The standardized regression coefficients between each item and the corresponding latent factors satisfied the significance level criterion. The values of the fitting indexes CFI, IFI, TLI, GFI, and RMSEA were 0.914, 0.928, 0.916, 0.891, and 0.051 (Table 4). These values indicate that the model fitted the observed data. The significance test of the path coefficient (standardized regression coefficient) in Figure 1 shows that PS has a significant direct positive effect on AP in statistics courses, with a standardized regression coefficient of 0.305 (*p* < 0.01). In contrast, PE has a significant direct negative effect on AP, with a standardized regression coefficient of −0.387 (*p* < 0.01) (Table 5). The direct influence of CM, DA, and OR on AP was insignificant, with respective *p*-values of 0.375, 0.584, and 0.717. There was thus insufficient evidence for a relationship between CM and AP, which is consistent with previous research in other academic fields. Except for OR, correlations between latent factors and AP were significant (*p* < 0.01).

**TABLE 4:** Fitting indexes of the revised Chinese structural equation model SEM-FMPS-I and the simplified revised Chinese structural equation model SEM-FMPS-II (*n* = 212).

Fitting index	SEM-FMPS-I	SEM-FMPS-II	Criterion
χ^2^	472.38	35.24	
*df*	258	24	
χ^2^/*df*	1.831	1.468	<3.0
TLI	0.916	0.967	≥0.90
GFI	0.891	0.965	≥0.80
CFI	0.914	0.978	≥0.90
IFI	0.928	0.978	≥0.90
RMSEA	0.051	0.042	<0.08
90% CI RMSEA	0.043–0.061	0.000–0.078	

The criteria of adequacy used to evaluate the models are from Arpaci and Baloğlu (2016).

**TABLE 5:** Regression coefficients between academic performance in statistics courses and latent factors in the structural equation model SEM-FMPS-I.

Relationship	Standardized regression coefficient	Non-standardized regression coefficient	S.E.	C.R.	*p*
PE→AP	−0.387	−7.311	1.822	−4.014	<0.001**
DA→AP	0.068	1.455	2.659	0.547	0.584
PS→AP	0.305	6.393	2.181	2.931	0.003**
OR→AP	0.030	0.813	2.241	0.363	0.717
CM→AP	−0.121	−1.936	2.180	−0.888	0.375

**Significant correlation at the 0.05 level (two-tailed). CM, concern over mistakes; DA, doubts about actions; PS, personal standards; PE, parental expectations; OR, organization; AP, academic performance in statistics courses.

**FIGURE 2:**
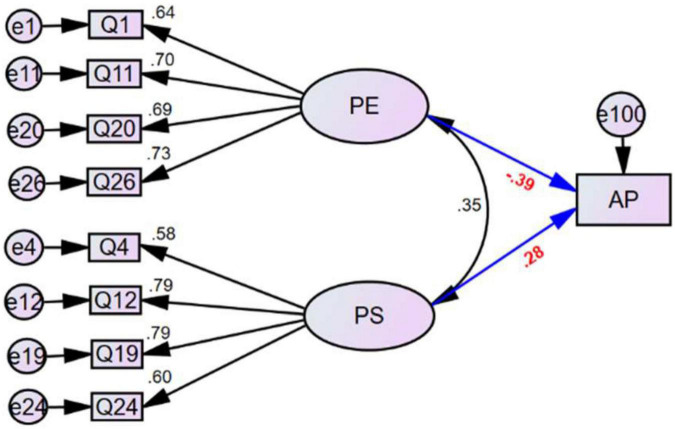
Final (simplified) model, SEM-FMPS-II, of the factors of perfectionism and their relationships with academic performance. PE, parental expectations; PS, personal standards; AP, academic performance in statistics courses.

We derived a simplified model, SEM-FMPS-II, from SEM-FMPS-I based on the preceding analysis to determine the strength and significance of the relationship between perfectionism and AP by removing latent factors that did not show a significant relationship in SEM-FMPS-I (CM, DA, and OR; Figure 2). The model fitting indexes of the simplified model showed acceptable model fitting (χ^2^/*df* = 1.468, CFI = 0.978, IFI = 0.978, TLI = 0.967, GFI = 0.965, RMSEA = 0.042; Table 4). There was no substantial change in the importance or magnitude of the relationships between PE or PS and AP in SEM-FMPS-II. Of concern, however, was the nature and direction of the relationships of PS and PE with AP. Although the relationship between PS and AP was opposite to that of PE, the relationships of PS and PE with perfectionism were still positive (*r* = 0.355, *p* < 0.001).

### The relationships of statistics anxiety and self-perception with academic performance

The Mann–Whitney *U*-test was performed on the scores of various factors of statistics anxiety grouped by gender. The results showed that the scores of female students were significantly higher than those of male students for the TCA factor (*Z* = −3.393, *p* = 0.001) and the FAH factor (*Z* = −2.439, *p* = 0.015). There was no significant difference in the other factors, WS (*Z* = −1.47, *p* = 0.142), FST (*Z* = −1.348, *p* = 0.178), and CS (*Z* = −1.817, *p* = 0.069) with respect to self-perception. The total score of female students in all factors was significantly higher than that of male students (*Z* = −2.686, *p* = 0.007). These results show that female students had a greater degree of statistics anxiety than male students.

**FIGURE 3:**
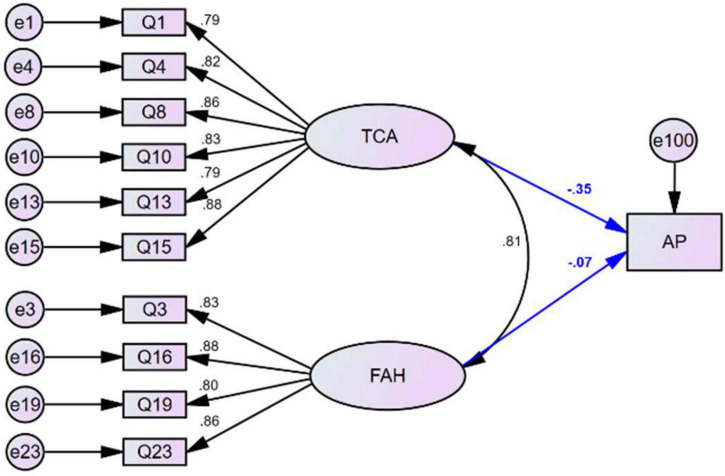
The structure of the SEM-SA model relating statistics anxiety with the two factors test and class anxiety (TCA) and fear of asking for help (FAH) and their relationship with academic performance (AP) in statistics courses.

**FIGURE 4:**
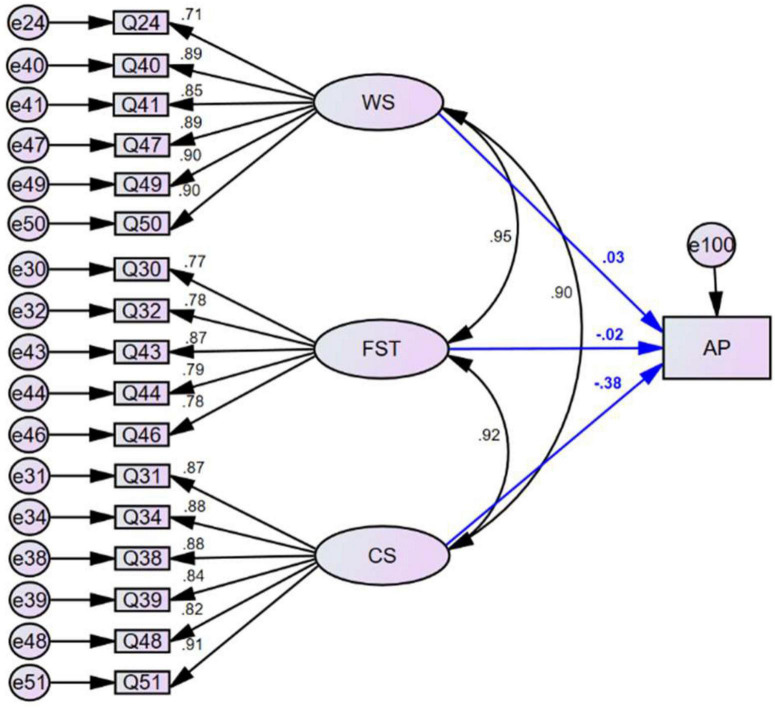
The structure of the SEM-SP model relating self-perception with the three factors worth of statistics (WS), fear of statistics teachers (FST) and computation self-concept (CS) and their relationship with academic performance in statistics courses (AP).

We created a statistics anxiety structural equation model (SEM-SA) (Figure 3) with AP as the dependent variable and the factors TCA and FAH as independent variables. We also created a self-perception structural equation model (SEM-SP) (Figure 4) with AP as the dependent variable and the factors WS, FST, and CS as independent variables. The fitting indexes for SEM- SA and SEM-SP (Table 6) all satisfied the criteria of adequacy for evaluation of the models. The significance test of the standardized regression coefficient showed that the factors TCA and CS each had a significant direct negative effect on the scores of statistics courses; the standardized regression coefficient for TCA was −0.350 (*p* < 0.01; Table 7) and for CS was −0.384 (*p* < 0.05; Table 8). The factors FAH (*p* = 0.540), WS (*p* = 0.916), and FST (*p* = 0.957) each had a non-significant direct influence on AP.

**TABLE 6:** The fitting indexes of the structural equation models SEM-SA and SEM-SP for the statistics anxiety rating scale STARS (*N* = 301).

Fitting index	SEM-SA	SEM-SP	Criterion
χ^2^	63.958	23.561	
*df*	36	119	
χ^2^/*df*	1.777	1.937	<3.0
TLI	0.983	0.957	≥0.90
GFI	0.964	0.903	≥0.80
CFI	0.989	0.966	≥0.90
IFI	0.989	0.967	≥0.90
RMSEA	0.051	0.059	<0.08
90% CI RMSEA	0.030–0.071	0.041–0.048	

The criteria of adequacy for evaluation of the model are from Arpaci and Baloğlu (2016). SEM-SA is the statistics anxiety structural equation model. SEM-SP is the self-perception structural equation model.

**TABLE 7:** Regression between academic performance in statistics courses and latent factors in the SEM-SA model.

Relationship	Standardized regression coefficient	Non-standardized regression coefficient	S.E.	C.R.	*p*
Test and class anxiety (TCA)→AP	−0.350	−0.434	0.136	−3.193	0.001**
Fear of asking for help (FAH)→AP	−0.067	−0.083	0.135	−0.613	0.540

**Significant correlation at the 0.05 level (two-tailed). AP, academic performance in statistics courses.

**TABLE 8:** Regression between academic performance in statistics courses and latent factors in the SEM-SP model.

Relationship	Standardized regression coefficient	Non-standardized regression coefficient	S.E.	C.R.	*p*
Worth of statistics (WS)→AP	0.028	0.041	0.391	0.106	0.916
Fear of statistics teachers (FST)→AP	−0.016	−0.020	0.373	−0.053	0.957
Computation self-concept (CS)→AP	−0.384	−0.462	0.195	−2.371	0.018**

**Significant correlation at the 0.05 level (two-tailed). AP, academic performance in statistics courses.

## Discussion

Our main objective in this study is to determine whether the dimensions of perfectionism are related to student AP (grades) in statistics courses. Previous studies have shown that PS have a direct positive effect on AP in statistics courses (Bieling et al., 2003), an outcome supported by the results of this study. PS for a perfectionist were defined as an individual tendency to set very high standards, reflecting an overemphasis on self-evaluation according to those very high standards (Frost et al., 1990). The relationship that we identified between perfectionism and AP in statistics courses supported the belief that hard work and high standards will improve grades in college courses.

Good communication between parents of acceptable and reasonable expectations and their children can influence the motives and aspirations of the children. Parental pressure on lifestyle and the physiological characteristics of college students tend to hinder communication between parents and children (Reti et al., 2002). Miscommunication can indirectly raise or lower PE and thus inhibit the development of motivation from achieving goals that meet PE (Su and Dong, 2015). Expectations are internalized and become motivators only when children perceive that their parents accept them as individuals and gain the understanding and ability to meet PE (Su and Dong, 2015).

Although parents expect their children to perform well, they may not be well integrated into their children’s student lives. Lack of parental judgment, support and direct participation in a child’s life can impede the child’s internalization of PE. PE that are moderate, reasonable, realistic and consistent with the child’s self-expectations will positively influence the child’s motivation to achieve goals that match those expectations. Expectations translated into goals that are too high or too low have a negative effect and are not well internalized by the child. The degree of internalization of PE directly affects a child’s ability to self-manage and therefore influences motivation to achieve goals (Li and Han, 2020).

Our results show that the direct effect of PE on AP is negative. An explanation is that children do not fully comprehend PE. They may be viewed as a form of harassment, resulting in resistance to their acceptance. However, there was a significant positive correlation between PE and the PS of college students, which suggests that PE can improve self-management and AP by influencing the PS of college students. Adaptive perfectionism is therefore moderately associated with AP, and we have shown that PS are an important mediator between perfectionism and AP.

Our analysis confirmed that the CM factor has nothing to do with AP, which is consistent with the studies of Seipel and Apigian (2005) and Siew and McCartney (2019). It is an important result. A focus on mistakes is recognized as maladaptive perfectionism. A negative response to mistakes is often interpreted as equating mistakes with failure, or a belief that failure will lead to loss of respect from others (Frost et al., 1990). Some studies have shown a significant positive correlation between the factors of CM and test anxiety (Song and Luo, 2017; Siew and McCartney, 2019). Our results indicate that test anxiety and statistical problem-solving anxiety significantly affect test scores. The factor of CM is internalized as statistics anxiety and self-perception and thus decreases test scores. College students usually regard statistics as a mathematical course, and their excessive subjective statistics anxiety will adversely affect their AP (Arpaci and Baloğlu, 2016).

The factor OR had no direct effect on AP. We attribute this finding to OR not being significantly related to test anxiety. The OR factor in FMPS does not measure the negative psychological quality like excessive pursuit of precision, order and OR that is defined by Frost et al. (1990) but rather measures an individual’s capacity to organize in pursuit of neatness (Zi, 2007). Excessive emphasis on accuracy, order and OR as arrangement is a critical and demanding tendency (Hollender, 1965) that was found to be the least relevant dimension of perfectionism (Frost et al., 1993). Our correlation analysis of the five factors of perfectionism supported this conclusion by showing that OR was significantly related to PS (*r* = 0.20, *p* < 0.05) but had no significant correlation with other factors, whereas there were significant pairwise correlations between other factors. The lack of a direct effect of OR on AP was in part ascribed to the direct effects of other factors of statistics anxiety on AP, reducing the direct effect of OR (Siew and McCartney, 2019).

We note a major limitation of this study. The perfectionism and statistics anxiety instruments were administered only to college students in one university in one city in China. Future research using college students from other regions of China as subjects is needed to obtain larger and more representative samples and to verify the robustness and generalizability of the findings of this study. The self-learning awareness of college students is particularly important in the context of online teaching during the COVID-19 pandemic. It is necessary to investigate whether PS influenced by perfectionism can be maintained to ensure learning. Future research should therefore examine the difference in statistics anxiety between online and traditional classroom instruction. Our findings suggest that appropriate adjustment of teaching methods will improve learning outcomes, and further assessment should be made to determine whether AP is improved when teaching practices are reformed to decrease statistics anxiety.

## Conclusion and practical implications

We demonstrated the direct effects of PS on AP in statistics courses in China. We also found PE were significantly related to PS, thereby having an indirect negative effect on AP. We found that other factors of perfectionism, such as CM, OR, and DA, did not directly affect AP. Statistics anxiety is a common reality in China, and there is a significant direct negative influence on AP by two factors of statistics anxiety, including TCA and CS.

Benefits will be gained from the preceding conclusions if their practical implications are addressed. The understanding of perfectionism, statistics anxiety, and self-perception in college students allows statistics instructors to improve their teaching practices and innovate in their statistics courses to enhance teaching effectiveness and better cultivate student talent. We suggest the following approaches.

### Reduce statistics anxiety and increase self-perception

Different intervention measures are required for different levels of statistics anxiety. For students who believe that statistics is useless, a detailed explanation of the wide application and value of statistics should be given, and the real-world importance of obtaining basic statistical literacy should be emphasized. Students who are afraid of mathematics or statistics, especially female students, may have their anxiety reduced if they are aware of the necessity of strong mathematics and statistics capability in getting a good grade in fields which require the application of statistics. This increased awareness can improve their self-perception of their ability to solve statistical problems and help them to overcome the anxiety they experience when confronted by statistical problems. Instructors must engage in timely classroom interventions to identify student personality traits and needs to help them overcome classroom and test anxiety, thus achieving the goal of improving their scores and increasing their ability to apply statistics.

### Actively internalize parental expectations and raise personal standards

Ideological and political elements can be incorporated into teaching at the appropriate time; the personal growth of famous scholars and teachers in China and the importance of parents and family in education can be introduced. Students can be encouraged to take the initiative in communicating to their parents their own ideas about their personal development, life plans and future expectations. Parents can also be guided to set reasonable expectations for their children and to identify appropriate and practical goals in order to enable college students in self-management and developing PS to positively affect students’ AP.

### A constructivist approach and innovation with teaching methods

Statistics instructors should draw on the basic principles of statistics to build a framework that bridges statistical principles and statistical methods and links different statistical methods as a basis for understanding the content and OR of statistics. They should fully interpret the framework to students. The learning objectives of students must be clearly defined. The application of statistical methods in scientific research and practice and in business can be used as examples to reveal the basic principles and practical significance of statistical methods. Students can restructure their existing knowledge to accommodate new knowledge in response to a constructivist approach in classroom teaching practices. This will enable them to overcome statistics anxiety, increase self-expectation, and raise PS.

## Data availability statement

The raw data supporting the conclusions of this article will be made available by the authors, without undue reservation.

## Ethics statement

Ethical review and approval was not required for the study on human participants in accordance with the local legislation and institutional requirements. Written informed consent was obtained from all participants for their participation in this study.

## Author contributions

All authors listed have made a substantial, direct, and intellectual contribution to the work, and approved it for publication.
